# Supplementary figures and images for: Stress-induced changes in cognitive function and intestinal barrier integrity can be ameliorated by venlafaxine and synbiotic supplementations
Source: PeerJ. 2024 Feb 28;12:e17033. doi: 10.7717/peerj.17033 (PMC10908264; doi:10.7717/peerj.17033)

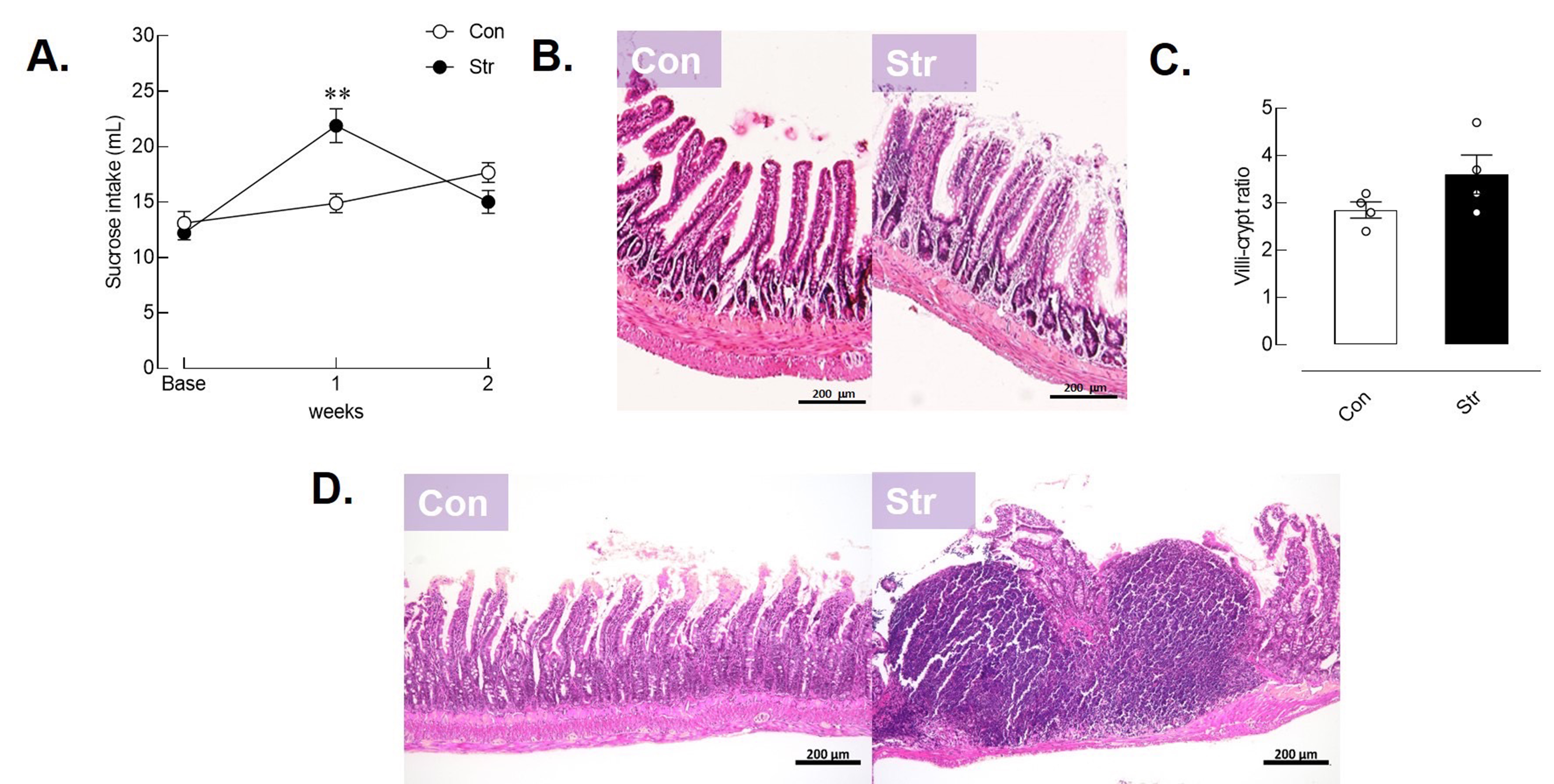

Supplement: Supplemental Information 3 — Weekly 2% sucrose intake test (n = 9 rats/group) (A), duodenal histology (B), villi-to-crypt ratio in duodenum (C), and characterization of Peyer’s Patches in ileum (D) (n = 4 rats/group). Con: control; Str: stressed+vehicle. [file peerj-12-17033-s003.jpg]

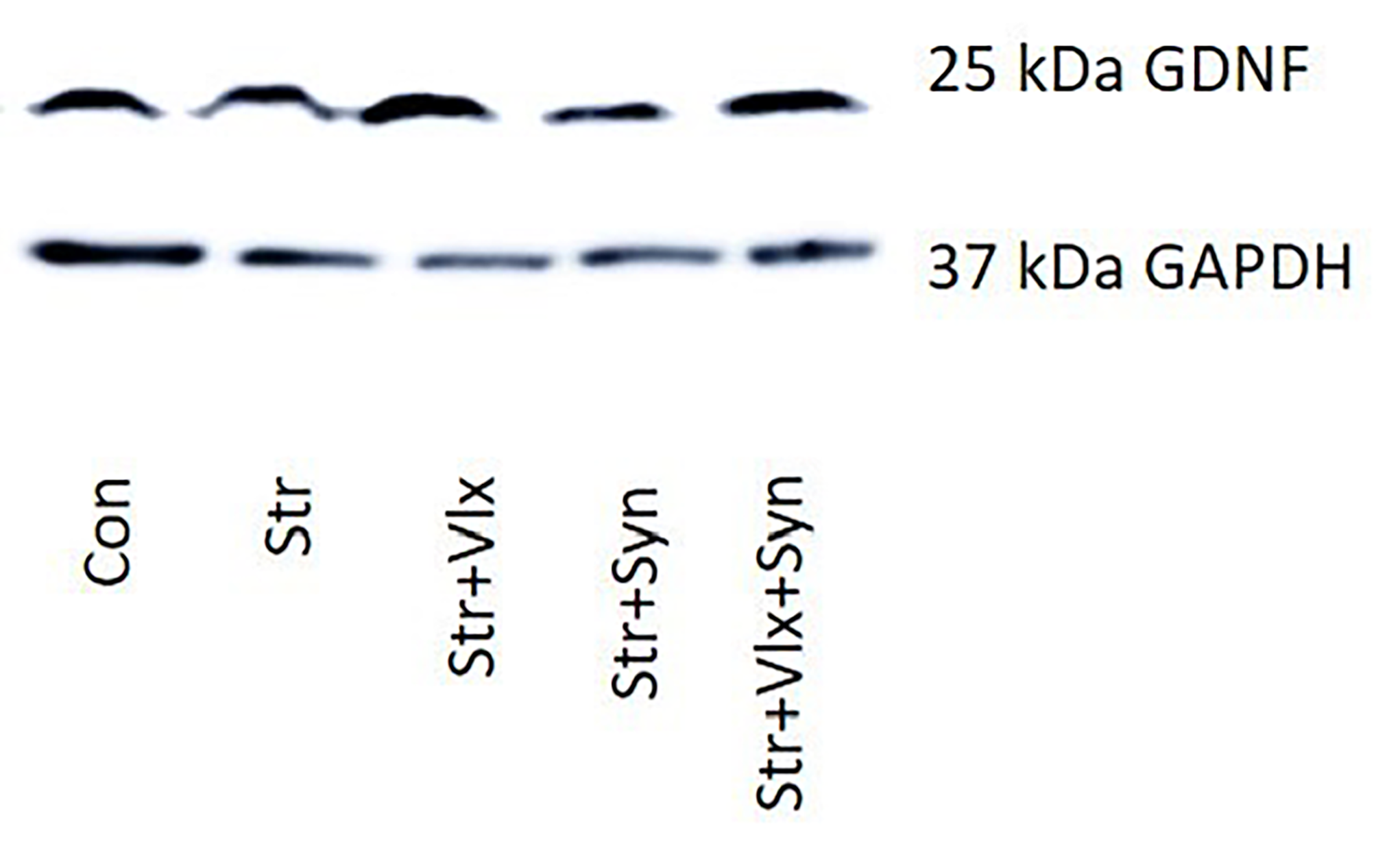

Supplement: Supplemental Information 4 [file peerj-12-17033-s004.tiff]

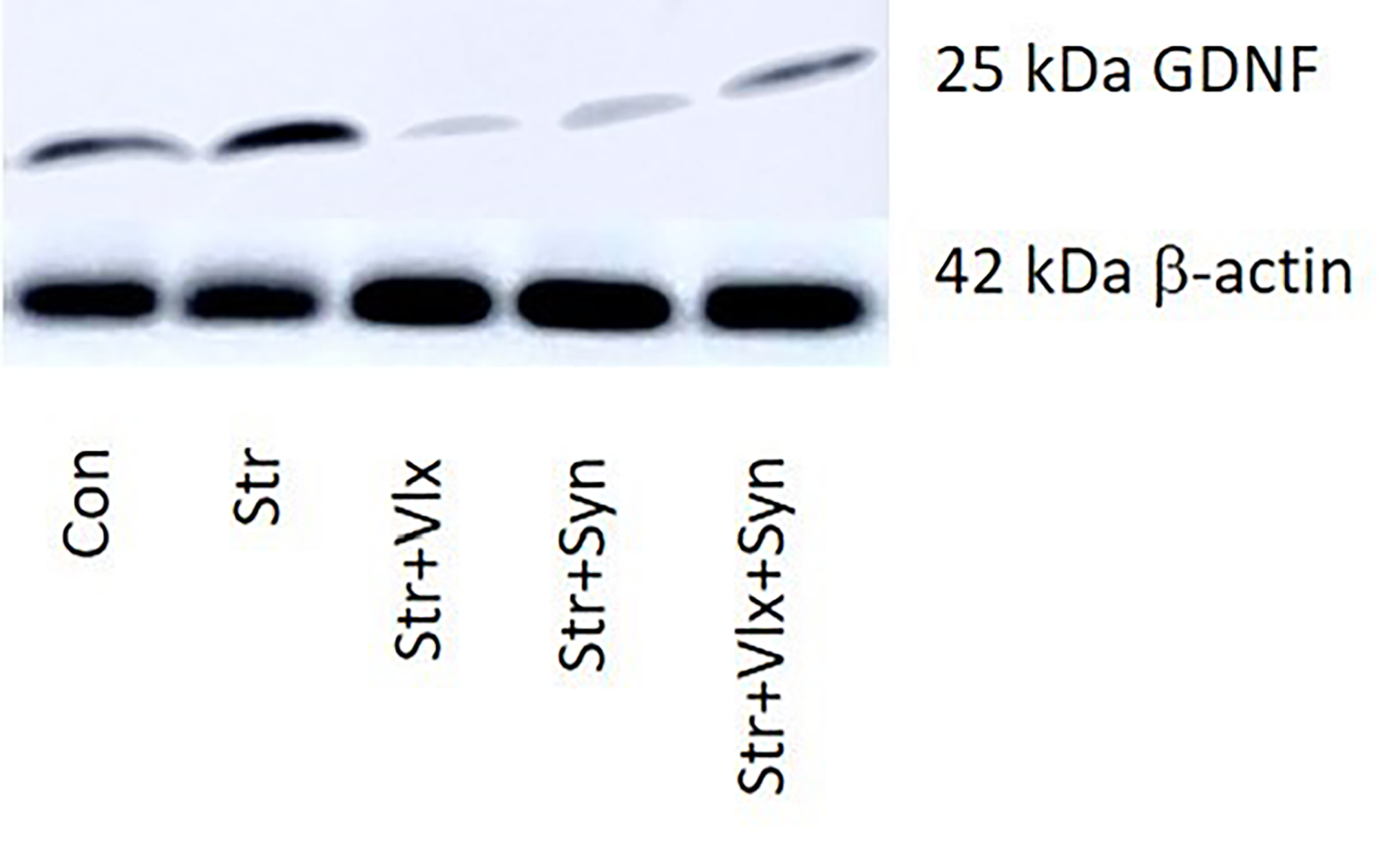

Supplement: Supplemental Information 5 [file peerj-12-17033-s005.tiff]
